# From bench to bedside: ^64^Cu/^177^Lu 1C1m-Fc anti TEM-1: mice-to-human dosimetry extrapolations for future theranostic applications

**DOI:** 10.1186/s13550-023-01010-4

**Published:** 2023-06-14

**Authors:** Silvano Gnesin, Nicolas Chouin, Michel Cherel, Steven Mark Dunn, Niklaus Schaefer, Alain Faivre-Chauvet, John O. Prior, Judith Anna Delage

**Affiliations:** 1 Institute of Radiation Physics, Lausanne University Hospital and University of Lausanne, 1011 Lausanne, Switzerland; 2 Inserm, CNRS, University of Angers, Oniris, CRCI2NA, University of Nantes, Nantes, France; 3 CHU Nantes, CNRS, Inserm, CRCINA, University of Nantes, 44000 Nantes, France; 4 LAbCore, Ludwig Institute for Cancer Research, Lausanne University Hospital and University of Lausanne, 1066 Epalinges, Switzerland; 5 Department of Nuclear Medicine and Molecular Imaging, Lausanne University Hospital and University of Lausanne, Rue du Bugnon 46, 1011 Lausanne, Switzerland; 6 Radiopharmacy Unit, Department of Pharmacy, Lausanne University Hospital and University of Lausanne, 1011 Lausanne, Switzerland

**Keywords:** Theranostic, Fusion protein antibody, Tumor endothelial marker 1, Copper-64, Lutetium-177, PET imaging, Radioimmunotherapy, Dosimetry, Dose extrapolation, Clinical translation

## Abstract

The development of diagnostic and therapeutic radiopharmaceuticals is an hot topic in nuclear medicine. Several radiolabeled antibodies are under development necessitating both biokinetic and dosimetry extrapolations for effective human translation. The validation of different animal-to-human dosimetry extrapolation methods still is an open issue. This study reports the mice-to-human dosimetry extrapolation of ^64^Cu/^177^Lu 1C1m-Fc anti-TEM-1 for theranostic application in soft-tissue sarcomas. We adopt four methods; direct mice-to-human extrapolation (M1); dosimetry extrapolation considering a relative mass scaling factor (M2), application of a metabolic scaling factor (M3) and combination of M2 and M3 (M4). Predicted in-human dosimetry for the [^64^Cu]Cu-1C1m-Fc resulted in an effective dose of 0.05 mSv/MBq. Absorbed dose (AD) extrapolation for the [^177^Lu]Lu-1C1m-Fc indicated that the AD of 2 Gy and 4 Gy to the red-marrow and total-body can be reached with 5–10 GBq and 25–30 GBq of therapeutic activity administration respectively depending on applied dosimetry method. Dosimetry extrapolation methods provided significantly different absorbed doses in organs. Dosimetry properties for the [^64^Cu]Cu-1C1m-Fc are suitable for a diagnostic in-human use. The therapeutic application of [^177^Lu]Lu-1C1m-Fc presents challenges and would benefit from further assessments in animals’ models such as dogs before moving into the clinic.

**Supplementary Information:**

The online version contains supplementary material available at 10.1186/s13550-023-01010-4.

## Introduction

TEM-1, also named endosialin or CD248, is a transmembrane cell surface glycoprotein expressed on pericytes and fibroblasts during tissue development, tumor neovascularization and inflammation [1]. In normal adults, TEM-1 protein expression appears to be limited to normal endometrial stroma and occasional fibroblasts [1, 2]. In pathological indications, the presence of TEM-1 has been reported in a wide variety of cell types associated with malignant diseases [3] and clinical studies have shown a direct correlation between TEM-1 transcript levels and patients outcomes with elevated levels of TEM-1 associated with nodal involvement and disease progression [4, 5]. Based on its pattern of expression and its association with pathology, TEM-1 is considered by the research community as a promising target [6, 7].

In humans, it has been recently reported that soft tissue sarcomas (STS) express TEM-1 with a high level of staining (96% of expression) [8]. Sarcoma appears as an attractive target as TEM-1 is simultaneously expressed in the TEM-1 vasculature, stroma and tumor cells [9]. Currently, the medical care for STS comprises surgery for local disease, radiotherapy and chemotherapy, but the prognosis of patients with metastasis or unresectable tumor is poor, with an overall survival of less than 50% at 5 years for advanced stages [10]. The development of new therapeutic strategies with new classes of molecules is thus needed.

Radiotheranostics is a rapidly-evolving branch of theranostics, with emerging opportunities for the personalization of therapy, facilitating potential improvements in patient care and clinical outcome. Nowadays, radiotheranostics is at a tipping point, and is moving into the mainstream of cancer therapeutics [11]. Theranostics radiopharmaceuticals carry alpha or beta emitters to the target tissues by attaching ligands such as small molecules, peptides or antibodies, to chelators that complex radioisotopes for systemic delivery.

We previously developed and tested preclinically a fully human single-chain variable fragment (scFv)-Fc fusion, 1C1m-Fc, cross-reacting with both murine and human TEM-1. This fusion protein antibody was conjugated with DOTA and radiolabeled with ^64^Cu for PET imaging [12] and with ^177^Lu for therapeutic purposes [13, 14]. The antibody biodistribution and imaging contrast were improved and our radiolabeled fusion protein antibody was validated as a potential theranostic tool to target TEM-1 with high quality PET/CT images and promising therapeutic applications.

Small animal data are essential to obtain successful clinical translation of new radiopharmaceuticals. Similarly, response and toxicity prediction are key steps for the implementation of a new theranostic agent. In contrast to chemotherapy or biologic treatments, the radiation delivery and the biological response to radiation can be modeled mathematically and used to understand the parameters of the treatment that are most important in influencing efficacy and toxicity [15]. The absorbed dose (AD), defined as the energy absorbed per unit of mass of tissue, mediated the biological effects of radionuclide therapy may be used to predict biological response [16]. In a preclinical study, the assessment of the AD to target tissues, based on biokinetic data, is usually obtained from the measurement of the *ex-vivo* activity present in dissected source organs performed at multiple time points after the injection of the radiopharmaceutical in a cohort of a specific animal model.

In view of a future transfer of our preclinical results obtained in mice with the tested radiolabeled fusion protein antibody to a first-in-human clinical study for STS disease, it is mandatory to perform the animal-to-human radiation dosimetry translation by extrapolating the observed murine data to the human model [17, 18]. Due to the difference in size organ masses in relation to the whole-body weight, and metabolic rates across the animal species (mice and the human in this case), reference dosimetry extrapolation methods have been developed [17, 19]. Unfortunately, the reliability and validation of these different extrapolation approaches are rarely documented in the published literature [18]. Hence, animal to human dosimetry extrapolation remains a matter of further study and development.

The aim of this study is to present and discuss the first extrapolation of AD to humans from previously obtained mice data to support the possible theranostic application of the TEM-1 compound. We applied reference computational methods of animal-to-human AD extrapolation as recently reported by Cicone et al. [19].

## Materials and methods

### Radiopharmaceutical

A single-chain variable fragment (scFv)-Fc fusion, named 1C1m-Fc (Molecular Weight = 106,196.8 Da), synthetized at the LAbCore immunoglobulin discovery and engineering facility, Ludwig Institute for Cancer Research Lausanne, was used. This fusion protein antibody has the properties to bind to the extracellular domain of both human and murine TEM-1 antigen and has been described previously [13, 20].

The conjugation and radiolabeling method as well as the different steps of the preclinical evaluation of the 1C1m-Fc fusion protein antibody, radiolabeled either with 64Cu (half-life of 12.7 h) or 177Lu (half-life of 6.6 days) was described earlier in Delage et al. [12–14].

### Biodistribution study

Murine xenograft model was described in Delage et al. [13]. Biodistribution studies were performed in female Balb/c nude mice (Charles River Laboratories, Wilmington, MA, USA) aged from 6 to 10 weeks. Methodology of the biodistribution studies was described in our previous articles [12–14].

The biodistribution data, based on ex-vivo source organ measurements in a calibrated gamma counter, obtained with [^177^Lu]Lu-1C1m-Fc conjugated with 1 and 3 DOTA at 4, 24, 48, 72 h and 6 days (*n* = 3 per timepoint) [13, 14] and with [^64^Cu]Cu-1C1m-Fc conjugated with 3 DOTA at 4 h (*n* = 4), 24 h (*n* = 5) and 48 h (*n* = 5) [12] were used for the dosimetry extrapolation.

### Dosimetry

We computed the time-integrated activity coefficients (TIACs) for the three radiolabeled compounds by time integration of normalized time activity curves (nTAC) as reported in [12–14]. Source organ TIACs, corrected for tumor sink effects [21], were computed for the average nTACs and the nTACs obtained by fitting the average time activity data ± 1SD, hence providing an upper/lower TIAC values defining the range of variability of each source organ TIAC for each radiolabeled compound.

We obtained mice-to-human TIAC extrapolations according to four methods as described and applied in previous literature [19, 22].

Method 1 (M1) considered a direct TIAC extrapolation from mice (TIAC_m_) to human (TIAC_h_).

1 $${\text{TIAC}}_{{\text{h,M1}}} \left( {{\text{organ}}} \right) = {\text{TIAC}}_{{\text{m}}} \left( {{\text{organ}}} \right)$$

Method 2 (M2) applied a corrective scaling factor to the mice organ source organ TIAC to consider the relative mass ratios between the considered source organ masses (m(organ)) and the whole-body masses (WB) in mice and humans.

2 $${\text{TIAC}}_{{\text{h,M2}}} \left( {{\text{organ}}} \right) = {\text{TIAC}}_{{\text{m}}} \left( {{\text{organ}}} \right) \times \left( {\frac{{{\text{m}}({\text{organ}})_{{\text{h}}} /{\text{WB}}_{{\text{h}}} }}{{{\text{m}}({\text{organ}})_{{\text{m}}} /{\text{WB}}_{{\text{m}}} }}} \right)$$

In Method 3 (M3), the human source organ TIACs were obtained by mono-exponential time integration of murine nTAC where the organ specific biological half-life (□_b_) was scaled to consider the different metabolic rate between species.

3 $${\text{TIAC}}_{{\text{h,M3}}} \left( {{\text{organ}}} \right) = \frac{{C_{{\text{m,organ}}} \left( {t = 0} \right)}}{{k_{{\text{b}}}^{ - 1} \times \lambda_{{\text{b}}} + \lambda_{{\text{p}}} }}$$

More detail about the application of this method can be found in previous publications [19, 23].

Method 4 (M4) combined the relative mass scaling applied in M2 and the metabolic scaling used in M3.

4 $${\text{TIAC}}_{{\text{h,M4}}} \left( {{\text{organ}}} \right) = {\text{TIAC}}_{{\text{h,M3}}} \left( {{\text{organ}}} \right) \times \left( {\frac{{{\text{m}}\left( {{\text{organ}}} \right)_{{\text{h}}} /{\text{WB}}_{{\text{h}}} }}{{{\text{m}}\left( {{\text{organ}}} \right)_{{\text{m}}} /{\text{WB}}_{{\text{m}}} }}} \right)$$

Relative mass scaling applied in M2 and M4 considered source organ masses in murine models as reported in previous literature [12–14] and the source organ human masses of the adult male and female ICRP-89 models [24].

For the three considered radiolabeled compounds, extrapolated human source organ TIAC (average, upper and lower values) were used as input for the kinetic model in the OLINDA/EXM 2.1 software (HERMES Medical Solution AB, Stockholm, Sweden) [25]. The software computed the target organ absorbed doses (average, upper and lower AD) and effective doses (E) for the male, female and the gender averaged adult human subjects using the formalism indicated in the ICRP-103 publication [26].

To obtain predictions of the amount of activity to administer in human patients in view of a possible therapeutic translation of the ^177^Lu radiolabeled compounds, we computed the maximum tolerable cumulated activity compatible with the levels of toxicity in possible limiting organs such as the heart wall, the kidneys, the liver, the lungs, the red-marrow, and the uterus. We considered AD toxicity thresholds from data available for radiopharmaceutical therapy for nuclear medicine procedures. In case of missing data, toxicity thresholds accepted for external beam irradiation therapy (EBRT) were used instead. Reference safety AD limits for thoracic organs such as the heart wall (30 Gy) and the lungs (20 Gy) were from Kong et al. [27]. The 40 Gy considered for a total liver irradiation comes from the toxicity level adopted in ^90^Y selective internal radiation therapy [28]. In this work we assumed a maximum tolerable AD of 30 Gy to the kidneys, which is commonly used in therapeutic applications of ^177^Lu by radiologists, even if a clear dose/safety threshold has not been yet established and reasonable toxicity limits are beyond this assumed AD level [29]. The safety limits of 16 Gy to the uterus was derived from EBRT and considers the possibility to maintain the fertility in young female patients [30]. Lastly the 2 Gy and 4 Gy safety limits for the red-marrow and the whole body are adopted from the ^131^I [31] and [^131^I]-I-MIBG [32] therapy respectively. The AD threshold of 60 Gy to obtain tumor control in STS was taken from EBRT [33]. In particular, to compute the activity required to reach the target tumor dose, we considered the tumor to normal liver AD ratio of 1.4 obtained for the [^177^Lu]Lu-1C1m-Fc 1 DOTA configuration [14]. This calculation applies only to M1 for which the 1.4 tumor-to-liver ratio was computed.

For sake of comparison, we compared target organ AD obtained for the diagnostic [^64^Cu]Cu-1C1m-Fc with two other diagnostic radiolabeled antibodies already tested in human, the [^64^Cu]Cu-DOTA-Trastuzumab [34] and the [^89^Zr]Zr-cmAb U36 [35] respectively.

## Results

For the four dosimetry methods described above (M1-M4), we reported the average values of source organ TIACs extrapolated to the human from previously published mice data for the [^177^Lu]Lu-1C1m-Fc conjugated to 1 DOTA (Table 1), and 3 DOTA (Table 2) and the [^64^Cu]Cu-1C1m-Fc conjugated with 3 DOTA (Table 3). Full TIAC data including the inf/sup values are provided in the Additional file 1: Tables S1, S2 and S3.

**Table 1:** Mice-to-human extrapolated dosimetry for [^177^Lu]Lu-1C1m-Fc 1 DOTA

[^177^Lu]Lu-1C1m-Fc 1 DOTA	TIAC (MBq.h/MBq)							
	M1		M2		M3		M4	
Source organs	M	F	M	F	M	F	M	F
Left colon	0.708	0.697	0.154	0.188	1.134	1.118	0.247	0.301
Small intestine	3.548	3.548	0.747	0.800	5.499	5.499	1.158	1.240
Stomach cont	0.824	0.824	0.324	0.365	0.824	0.824	0.324	0.365
Right colon	0.944	0.945	0.205	0.254	1.513	1.515	0.329	0.407
Rectum	0.456	0.465	0.099	0.125	0.731	0.745	0.159	0.200
Heart cont	1.961	1.961	2.436	2.150	5.364	5.364	3.455	3.050
Heart wall	0.509	0.509	0.402	0.371	1.066	1.066	0.843	0.777
Kidneys	3.592	3.592	0.919	0.992	4.928	4.928	1.261	1.361
Liver	22.894	22.894	9.183	8.690	22.401	22.401	8.985	8.503
Lungs	1.101	1.101	2.166	2.087	2.507	2.507	4.931	4.749
Ovaries		0.468		0.036		0.598		0.049
Salivary glands	0.800	0.852	0.161	0.161	0.800	0.852	0.161	0.172
Red marrow	2.211	2.475	5.588	5.230	2.922	3.070	7.386	6.912
Spleen	1.132	1.132	0.454	0.478	1.476	1.476	0.591	0.623
UB	0.186	0.199	0.677	0.624	0.186	0.199	0.677	0.665
Uterus		2.886		0.620		2.886		0.661
Total body	111.1	111.1	111.1	111.1	111.1	111.1	111.1	111.1
Rest of body	70.2	66.5	87.6	87.9	59.7	56.0	80.6	81.0

[^177^Lu]Lu-1C1m-Fc 1 DOTA mice-to-human extrapolation of average source organ TIACs was obtained with the four considered dosimetry methods (M1-M4) for both the adult male (M) and female (F) human subjects

**Table 2:** Mice-to-human extrapolated dosimetry for [^177^Lu]Lu-1C1m-Fc 3 DOTA

[^177^Lu]Lu-1C1m-Fc 3 DOTA	TIAC (MBq.h/MBq)							
	M1		M2		M3		M4	
Source organs	M	F	M	F	M	F	M	F
Left colon	0.609	0.600	0.130	0.158	0.985	0.970	0.210	0.255
Small intestine	2.842	2.842	0.570	0.755	4.521	4.521	0.906	0.970
Stomach cont	0.575	0.575	0.636	0.716	0.553	0.570	0.612	0.710
Right colon	0.651	0.687	0.139	0.181	1.313	1.315	0.280	0.346
Rectum	0.113	0.141	0.201	0.223	0.635	0.647	0.135	0.170
Heart cont	1.083	1.083	0.764	0.835	2.841	2.815	2.003	1.752
Heart wall	0.343	0.343	0.157	0.179	0.891	0.891	0.407	0.375
Kidneys	2.129	2.129	0.553	0.597	4.226	4.226	1.098	1.185
Liver	31.678	31.678	12.321	11.659	31.678	31.678	12.321	11.659
Lungs	0.744	0.744	1.646	1.964	1.894	1.894	4.191	4.037
Ovaries		0.241		0.013		0.448		0.024
Red marrow	2.508	2.635	2.508	2.635	6.517	6.847	6.517	6.847
Spleen	1.163	1.163	0.536	0.565	1.163	1.163	0.536	0.565
UB	0.140	0.140	0.140	0.140	0.140	0.140	0.140	0.140
Uterus		1.727		0.581		0.581		0.581
Total body	98.8	98.8	98.8	98.8	98.8	98.8	98.8	98.8
Rest of body	54.2	52.1	78.5	77.6	41.4	40.1	69.4	69.2

[^177^Lu]Lu-1C1m-Fc 3 DOTA mice-to-human extrapolation of average source organ TIACs was obtained with the four considered dosimetry methods (M1-M4) for both the adult male (M) and female (F) human subjects

**Table 3:** Mice-to-human extrapolation for [^64^Cu]Cu-1C1m-Fc 3 DOTA

[^64^Cu]Cu-1C1m-Fc 3 DOTA	TIAC (MBq.h/MBq)							
	M1		M2		M3		M4	
Source organs	M	F	M	F	M	F	M	F
Left colon	0.091	0.090	0.055	0.067	0.073	0.072	0.048	0.058
Small intestine	0.434	0.434	0.181	0.194	0.296	0.296	0.124	0.132
Stomach cont	0.096	0.096	0.084	0.094	0.077	0.077	0.067	0.075
Right colon	0.122	0.122	0.073	0.090	0.098	0.098	0.064	0.079
Rectum	0.059	0.060	0.035	0.044	0.047	0.048	0.031	0.039
Heart cont	0.513	0.508	0.146	0.128	2.645	2.645	0.754	0.665
Heart wall	0.187	0.187	0.151	0.139	0.175	0.175	0.141	0.130
Kidneys	0.585	0.585	0.165	0.178	0.645	0.645	0.182	0.196
Liver	2.689	2.689	1.164	1.101	2.429	2.429	1.051	0.995
Lungs	0.338	0.338	0.684	0.658	0.415	0.415	0.840	0.809
Ovaries		0.056		0.005		0.050		0.005
Red marrow	1.176	1.236	1.176	1.236	1.853	1.947	1.853	1.947
Spleen	0.194	0.194	0.087	0.092	0.209	0.209	0.093	0.098
Uterus		0.088		0.024		0.176		0.047
Total body	13.7	13.7	13.7	13.7	13.7	13.7	13.7	13.7
Rest of body	7.3	7.1	9.7	9.7	4.8	4.5	8.5	8.5

[^64^Cu]Cu-1C1m-Fc 3 DOTA mice-to-human extrapolation of average source organ TIACs was obtained with the four considered dosimetry methods (M1-M4) for both the adult male (M) and female (F) human subjects

In Table 4 we report the Olinda generated target organ AD for the gender average adult human subject for the main irradiated organs for the ^177^Lu radiolabeled compound using the four extrapolation methods described above (M1-M4).

**Table 4:** AD to selected target organs for the [^177^Lu]Lu-1C1m-Fc 1 DOTA and 3 DOTA

[^177^Lu]Lu-1C1m-Fc (average) Absorbed dose (Gy/GBq)								
Target organ	M1		M2		M3		M4	
	1 DOTA	3 DOTA	1 DOTA	3 DOTA	1 DOTA	3 DOTA	1 DOTA	3 DOTA
Heart wall	0.37	0.23	0.36	0.15	0.89	0.58	0.59	0.35
Kidneys	1.09	0.66	0.30	0.19	1.48	1.28	0.41	0.34
Liver	1.30	1.79	0.51	0.68	1.27	1.79	0.50	0.70
Lungs	0.11	0.08	0.19	0.16	0.22	0.17	0.41	0.35
Ovaries	3.63	1.88	0.30	0.11	4.63	3.47	0.40	0.11
Red marrow	0.19	0.19	0.35	0.21	0.21	0.37	0.43	0.39
Spleen	0.72	0.73	0.30	0.35	1.35	0.74	0.39	0.35
Uterus	3.13	1.88	0.69	0.64	3.13	0.64	0.73	0.11
Total body	0.15	0.14	0.15	0.14	0.15	0.14	0.15	0.14
E (Sv/GBq)	0.27	0.26	0.18	0.18	0.34	0.36	0.22	0.22

The table reports average AD and the effective dose for the gender averaged human adult model obtained with the OLINDA/EXM 2.1 software for each tested dosimetry methods (M1-M4), for the [^177^Lu]Lu-1C1m-Fc 1 DOTA and 3 DOTA

Table 5 reports the averaged AD for the [^64^Cu]Cu-1C1m-Fc 3 DOTA and for sake of comparison AD from the [^64^Cu]Cu-DOTA-Trastuzumab [34] and the [^89^Zr]Zr-cmAb U36 [35] respectively.

**Table 5:** [^64^Cu] AD to selected target organs and comparison with previously published data

[^64^Cu]Cu-1C1m-Fc 3DOTA (average) AD (mGy/MBq)					AD (mGy/MBq)	
Target organ	M1	M2	M3	M4	[^64^Cu]Cu-DOTA-Trastuzumab [34]	[^89^Zr]Zr-cmAb U36 [35]
Heart Wall	0.11	0.06	0.33	0.12	0.16	
Kidneys	0.17	0.06	0.19	0.06	0.09	1
Liver	0.16	0.07	0.15	0.06	0.12	1.3
Lungs	0.04	0.06	0.05	0.07		0.79
Ovaries	0.38	0.04	0.34	0.04		
Red Marrow	0.06	0.06	0.08	0.09	0.04	0.08
Spleen	0.12	0.06	0.13	0.06	0.10	0.72
Uterus	0.10	0.03	0.18	0.06		
E (mSv/MBq)	0.05	0.04	0.05	0.04	0.03	0.6

The table reports average AD and the effective dose for the gender averaged human adult model obtained with the OLINDA/EXM 2.1 software for each tested dosimetry methods (M1-M4). Original data for the [^64^Cu] Cu-1C1m-Fc 3DOTA are compared with previously published dosimetry from the [^64^Cu]Cu-DOTA-Trastuzumab [34] and the [^89^Zr]Zr-cmAb U36 [35] respectively

**Fig. 1:**
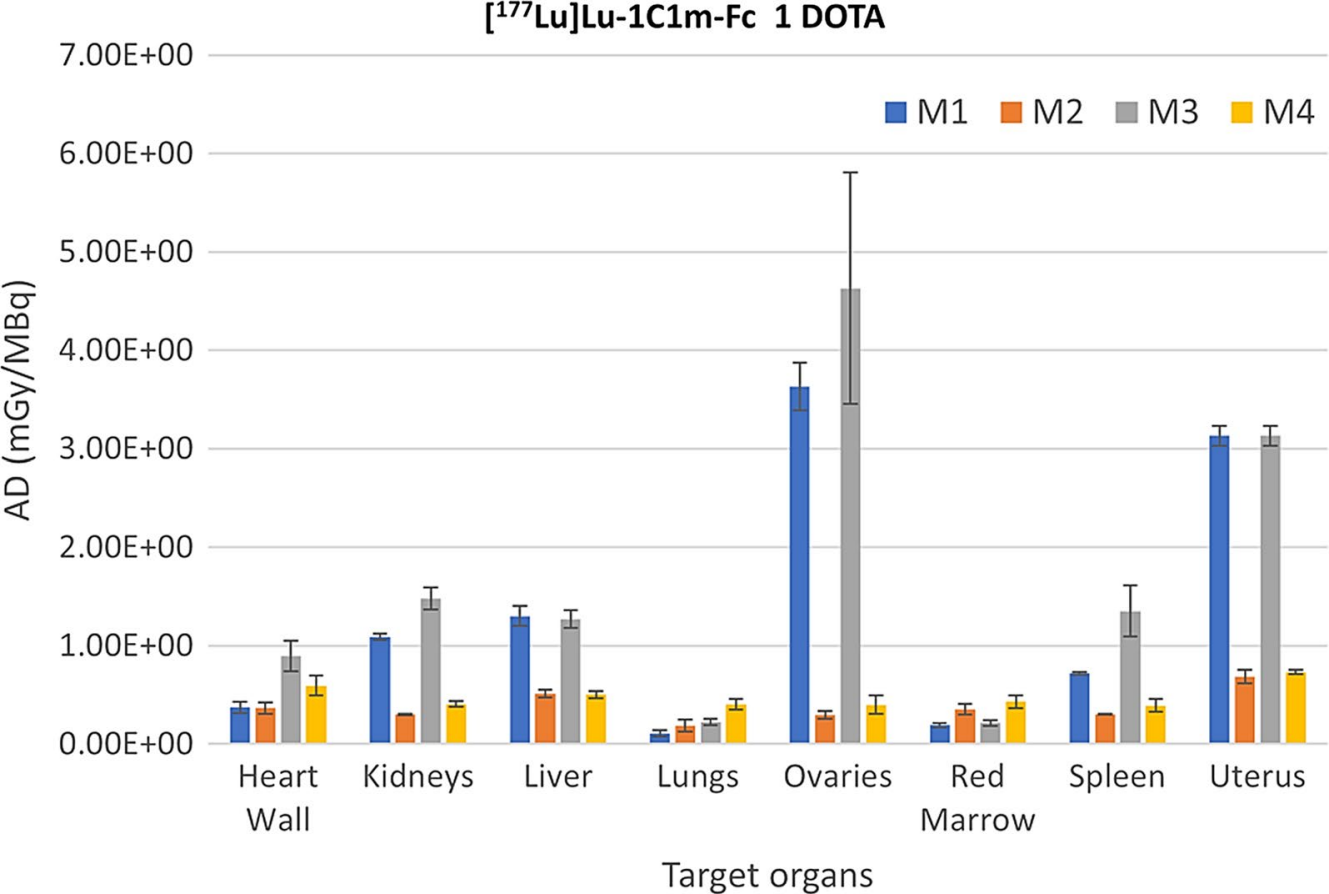
AD in target organs for the [^177^Lu]Lu-1C1m-Fc 1DOTA. The figure shows the average, upper and lower AD in target organs as a function of the dosimetry extrapolation method used. Bars indicated upper/lower AD

**Fig. 2:**
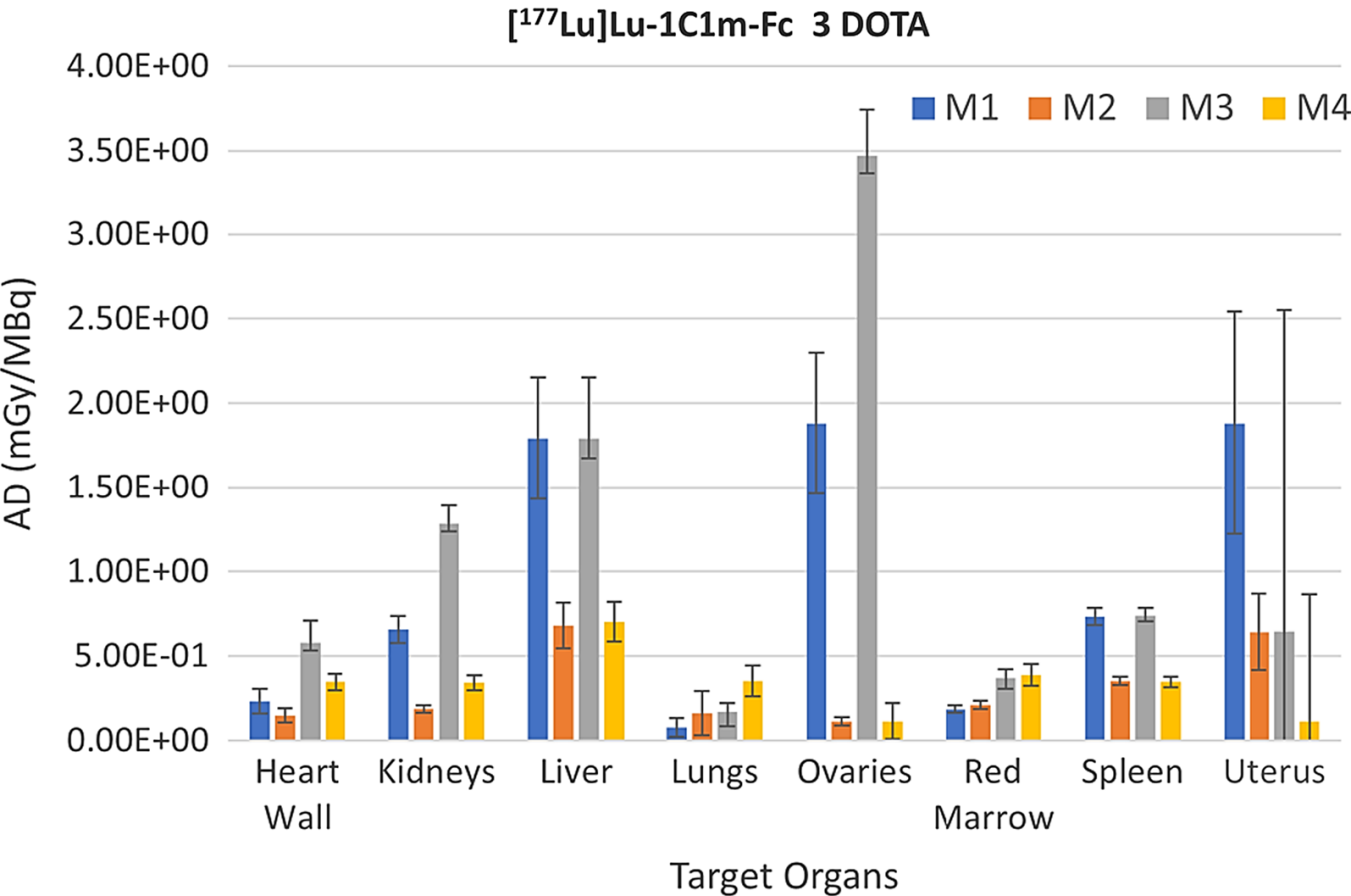
AD in target organs for the [^177^Lu]Lu-1C1m-Fc 3DOTA. The figure shows average, upper and lower AD in target organs as a function of the dosimetry extrapolation method used. Bars indicated upper/lower AD

In Figs. 1 and 2 we give a visual comparison of the target organ AD for the [^177^Lu]Lu-1C1m-Fc 1 DOTA and 3 DOTA respectively. This graphical representation gives the average AD with the upper/lower levels expressed by error bars.

The full data concerning AD in target organs for the male, female and gender averaged adult human subject including average, upper and lower values are reported in the Additional file 1: Tables S4, S5 and S6.

In Table 6 we reported the values of maximal administered activity compliant with the organ-specific AD safety limits reported in the materials and methods in prevision of a therapeutic use of the ^177^Lu radiolabeled compounds.

**Table 6:** Cumulated therapeutic activity to reach AD safety and efficacy levels

Target Organ (AD limit) [36]	Cumulated administered activity (GBq) to reach AD safety limit or AD efficacy threshold (in tumor)							
	M1		M2		M3		M4	
	1 DOTA	3 DOTA	1 DOTA	3 DOTA	1 DOTA	3 DOTA	1 DOTA	3 DOTA
Heart wall (30 Gy) [27]	80.4	129.3	82.4	204.1	33.6	51.8	50.7	87.0
Kidneys (30 Gy) [29]	27.5	45.8	100.3	161.3	20.3	23.4	74.1	88.0
Liver (40 Gy) [28]	30.8	22.3	78.3	58.7	31.5	22.3	79.8	57.0
Lungs (20 Gy) [27]	186.9	255.4	107.5	125.0	90.1	116.3	49.4	56.8
Red Marrow (2 Gy) [31]	10.5	10.8	5.7	9.6	9.4	5.4	4.7	5.2
Uterus (16 Gy) [30]	5.1	8.5	23.4	25.0	5.1	24.9	21.9	140.4
Total Body (4 Gy) [32]	25.5	28.8	25.5	28.8	25.5	28.8	25.5	28.8
Tumor STS (60 Gy) [33]	32.7	41.1						

The table reports the cumulated therapeutic administered activity required to reach limiting-organ AD safety limits and the efficacy AD thresholds of 60 Gy in STS for the [^177^Lu]Lu-1C1m-Fc 1 DOTA and 3 DOTA as a function of the mice-to-human dosimetry extrapolation method (M1-M4)

## Discussion

We have presented and compared the mice-to-human dosimetry extrapolation of ^177^Lu and ^64^Cu radiolabeled TEM-1 obtained with four reference methods. For the three considered radiolabeled TEM-1 compounds we reported important variation of AD to target organs as a function of the considered dose-extrapolation method. This aspect was previously discussed in Cicone et al. [19].

Furthermore, insufficient information is presently available in the literature to determine which animal-to-human dosimetry extrapolation method is the more appropriate for a specific type of radiolabeled molecule. Most of the publications reporting animal-to-human dosimetry extrapolations used only one method, typically M1 or M2.

As indicated in Figs. 1 and 2, we found that the application of M1 and M3, which did not consider organ mass scaling across species, resulted in significantly higher AD as compared to M2 and M4 in most considered target organs. This difference is more prominent in the female reproductive organs such as the uterus and the ovaries, for which AD obtained with M1 and M3 overestimate the AD obtained with M2 and M4 by a factor ranging from 4 to 10.

In particular, M4 takes into account two important factors of the inter-specie variability, such as the different metabolic rates, and the different relative organ masses compared to the individual whole body mass. Nevertheless, there is still poor scientific background to establish which of the tested methods for animal-to-human absorbed dose extrapolation would provide the better prediction. In most published literature, dosimetry extrapolation from pre-clinic setup to humans rely on the application of a single method and no systematic comparison with the in-human dosimetry is reported. Hence, based on the present knowledge we cannot argue for the superiority of one of the four tested methods. Future animal-to-human dosimetry comparison would hopefully provide more insight on this direction.

We observed that for many source organs TIACs (and hence AD) obtained by application of M2 and M4 presented similar values. The same is true for TIACs (and hence AD) obtained with M1 and M3.. This is particularly true for organs such as the liver, that have a relatively large biological half life comparable to the physical half life (for instance: 137 and ~ 160 h respectively for the case of Lu-177 TEM-1 1DOTA) and consequently the contribution of the metabolic correction was relatively small. Larger differences between M2 and M4 have been reported for instance for the cardiac wall in reason of the larger difference between the biological and physical half life (37 and 160 h respectively for the cas of the Lu-177 TEM-1 1DOTA).

We also observed, methods M2 and M4 produced systematically higher values of TIAC (and hence AD) than M1 and M3. This resulted from the important impact of the application relative organ mass rescaling correction for the mice-to-human case on study.

Considering a possible future therapeutic use of the ^177^Lu TEM-1 compounds, the AD estimates for target organs obtained with the different methods translate into different viable therapeutic cumulated activity administration levels that respect the safety limits in potentially critical organs (see Table 6).

From the results obtained in mice experiments, the [^177^Lu]Lu-1C1m-Fc conjugated with 1 DOTA presented the most favourable tumour-to-liver AD ratio of 1.4 [12]. Hence, we considered this form as an intesresting candidate for a possible in-human therapeutic translation for the treatment of STS patients. Accessing a solid tumor with a radiolabeled immuno-agent typically involves a long circulating half-life of the compound hence resulting in an important normal organ irradiation with a consequent, increased, haematological impact. This contrasts significantly with small peptides that are characterized by a rapid clearance and shorter tumour retention [16]. According to the obtained dose extrapolations for the human subject, the main constraint for the cumulated therapeutic administered activity of [^177^Lu]Lu-1C1m-Fc (1 DOTA) is determined by the safety dose limit of 2 Gy applied to the red-marrow. A single application of 5–10 GBq of this radiopharmaceutical would be sufficient to reach this safety limit. For the total body exposure, we retained a maximum acceptable dose of 4 Gy as indicated from the [^131^I]-I-MIBG treatment for neuroblastoma in pediatrics [32], where the 4 Gy safety limit was assumed as a surrogate estimation of the red-marrow AD exposure.

In our analyis we referred to organ AD safety limits from specific literature reports. However, it is reasonable to assume that, in the absence of a solid evidence-based background, such values may continue to evolve with the emergence of new findings. This is particularly relevant in the domain of radiophamaceutical therapies where, unlike for EBRT, a diversity of both targeting molecules and radioisotopes must be considered [37].

In our dosimetry extrapolations this level of AD for the total body is reached with 25 GBq of total cumulated therapeutic activity. Considering a target AD of 60 Gy in the tumoral volume (values assumed from EBRT for the response of STS [33]), the required therapeutic activity is about 30 GBq, a value approaching the safety limits in other important organs (other than the red-marrow) such as the liver and the kidneys. Considering the results obtained so far, and a 30 GBq therapeutic activity administration to achieve 60 Gy to the STS, this would require a patient specific dosimetric optimization of the treatment plan.

To avoid toxicity due to the myelosuppression-related toxicity and to allow escalation of the therapeutic activity administration to myeloablative levels, a number of strategies have been tested such as autologous marrow reinfusion or injection of granulocyte–macrophage colony-stimulating factor [38]. In the context of radioimmunotherapy with ^131^I, Press et al. [39] obtained a recovery of the number of neutrophils superior to 500/mm3 for a median (± SD) of 22 ± 9 days after marrow infusion, whereas platelet recovery was more variable, occurring at a median of 20 ± 27 days.

Moreover, fractionated therapeutic administration can be envisaged to decrease the heamatological toxicity to acceptable levels [40]. Since 1C1m-Fc is a fully human fusion protein antibody, we expect that it may be administered in repeated fractionated doses with a low immunogenicity risk and good predicted tolerance.

Special consideration should also be given to patient selection, in particular regarding any history of high dose chemotherapy which may increase the risk of developing bone marrow toxicity [41].

To explore the feasibility of a therapeutic approach based on the use of the [^177^Lu]Lu-1C1m-Fc in treating STS, further investigations in larger size mammals such as dogs and/or pigs could provide complementary useful information. Such animal models expressing TEM-1 positive STS will enable a more robust tumour-to-normal organ and red marrow dosimetry evaluation. In contrast to our previous studies in mice, in which red-marrow dosimetry was performed based on blood data only, in larger mammals the red marrow dosimetry could be improved by PET and SPECT imaging assessment that would complement and improve the estimation based on the blood sample collection. Furthermore, we stress the fact that the level of toxicity in many organs such as the liver, the kidneys and the red-marrow itself are not sufficiently characterized for the systemic administration of ^177^Lu labeled radiotherapeutics. This aspect is of key relevance and could be addressed in future ‘bridging’ studies in eg. dogs or pigs before progressing to in-human therapeutic applications. Dose-toxicity thresholds for ^177^Lu radioligands (^177^Lu-DOTA and ^177^Lu- PSMA) have not yet been defined [42–44]. Even the commonly accepted 2 Gy safety limits for the red marrow derives from historic data in ^131^I therapy [31], more recent dosimetric results increase this level to 3 Gy [45]. Previous experience with therapeutic administration of ^90^Y-Ibritumomab-Tiuxetan do not report correlations between bone marrow AD and hemato-toxicity for bone marrow AD of 2 Gy. Rather, the hemato-toxicity correlated with the ellapsed time from the last chemotherapy [41]. Moreover the AD threshold level for tumour response using ^177^Lu antibodies in STS is an unresolved issue that could be investigated with the escalation of the administered activity in such animal models.

In view of developping a full theranostic approach, we reported the mice-to-human extrapolation of the AD and E for the [^64^Cu]Cu-1C1m-Fc as a PET companion tracer of the [^177^Lu]Lu-1C1m-Fc. We compared AD estimations obtained from the four extrapolation methods with in-human dosimetric results reported in the literature for radiolabeled antibodies, namely: [^64^Cu]Cu-DOTA-Trastuzumab [34] and [^89^Zr]Zr-cmAb U36 [35].

[^64^Cu]Cu-DOTA-Trastuzumab was used in a clinical trial for PET/CT functional imaging of human epidermal growth factor receptor 2–positive metastatic breast cancer. Such a radiolabeled antibody presented similar effective dose and AD levels in targeted organs to the extrapolated human ADs reported by ourselves for the [^64^Cu]Cu-1C1m-Fc. The use of a ^64^Cu PET immuno-tracer is ideal for the short-term characterization of the uptake of the radiolabeled antibody particle in lesions within the two days after the diagnostic administration [34], thus providing key information for patient selection. However, because of the relatively short physical half-life (12.7 h), ^64^Cu PET immuno-tracers are not suited for pre-therapeutic dosimetry planning. [^89^Zr]Zr-cmAb U36 presented higher effective dose and higher AD level than our radiolabeled compound (Table 5). The use of ^89^Zr-radiolabeled immunotracers have shown promising results enabling the characterization of the biokinetics in tissues for a sufficiently long period of time (typically up to one week) [35]. Nevertheless, the relatively long half-life involves a less favorable dosimetry, limited availability of radionuclide, and patient convenience issues, since patients are required to return about 5 days after tracer administration [46].

## Conclusions

Dose estimation based on animal data is a mandatory requirement, providing important insights and guidance for predicting safety and efficacy of any radiolabeled compound prior to its diagnostic and/or therapeutic in-human translation. Different methods for animal-to-human dosimetry extrapolations have been reported in the literature but no consensus presently exists on the appropriate approach to apply for any specific radiolabeled compound. For the theranostic couple ^64^Cu/^177^Lu 1C1m-Fc anti TEM-1, we show an important variability in mice-to-human AD extrapolations depending on the method used. Furthermore, in considering a potential therapeutic application of [^177^Lu]Lu-1C1m-Fc, further dosimetry analysis supported by quantitative imaging investigations performed in a large mammal species bearing STS would prove highly informative.

## Supplementary Information

**Additional file 1: Table S1** TIAC for the [^177^Lu]Lu-1C1m-Fc 1 DOTA. **Table S2** TIAC for the [^177^Lu]Lu-1C1m-Fc 3 DOTA. **Table S3** TIAC for the [^64^Cu]Cu-1C1m-Fc 3 DOTA. **Table S4** Target organ AD for the [^177^Lu]Lu-1C1m-Fc 1 DOTA. **Table S5** Target organ AD for the [^177^Lu]Lu-1C1m-Fc 3 DOTA. **Table S6** Target organ AD for the [^64^Cu]Cu-1C1m-Fc 3 DOTA.

## Data Availability

The datasets used and/or analyzed during the current study are available from the corresponding author on reasonable request.
