# Supplementary material for: From bench to bedside: 64Cu/177Lu 1C1m-Fc anti TEM-1: mice-to-human dosimetry extrapolations for future theranostic applications
Source: EJNMMI Res. 2023 Jun 14;13:59. doi: 10.1186/s13550-023-01010-4 (PMC10267050; doi:10.1186/s13550-023-01010-4)
Supplement: Supplementary file 1 — Additional file 1: Table S1 TIAC for the [177Lu]Lu-1C1m-Fc 1 DOTA. Table S2 TIAC for the [177Lu]Lu-1C1m-Fc 3 DOTA. Table S3 TIAC for the [64Cu]Cu-1C1m-Fc 3 DOTA. Table S4 Target organ AD for the [177Lu]Lu-1C1m-Fc 1 DOTA. Table S5 Target organ AD for the [177Lu]Lu-1C1m-Fc 3 DOTA. Table S6 Target organ AD for the [64Cu]Cu-1C1m-Fc 3 DOTA. [file 13550_2023_1010_MOESM1_ESM.docx]

Supplementary materials

From bench to bedside: ^64^Cu/^177^Lu 1C1m-Fc anti TEM-1: mice-to-human dosimetry extrapolations for future theranostic applications

Table S1: TIAC for the [^177^Lu]Lu-1C1m-Fc 1 DOTA

**a)**

**b)**

**c)**

[^177^Lu]Lu-1C1m-Fc conjugated to 1 DOTA source organ TIACS; a) average TIAC; b) lower TIAC; c) upper TIAC values.

Table S2: TIAC for the [^177^Lu]Lu-1C1m-Fc 3 DOTA

**a)**

**b)**

**c)**

[^177^Lu]Lu-1C1m-Fc conjugated to 3 DOTA source organ TIACS; a) average TIAC; b) lower TIAC; c) upper TIAC values.

Table S3: TIAC for the [^64^Cu]Cu-1C1m-Fc 3 DOTA

**a)**

**b)**

**c)**

[^64^Cu]Cu-1C1m-Fc conjugated to 3 DOTA source organ TIACS; a) average TIAC; b) lower TIAC; c) upper TIAC values

Table S4: Target organ AD for the [^177^Lu]Lu-1C1m-Fc 1 DOTA

**a)**

**b) c)**

[^177^Lu]Lu-1C1m-Fc conjugated to 1 DOTA gender average human subject dosimetry (mGy/MBq or equivalently Gy/GBq); a) Average Absorbed Dose; b) Lower Absorbed Dose; c) Upper Absorbed Dose values.

Table S5: Target organ AD for the [^177^Lu]Lu-1C1m-Fc 3 DOTA

**a)**

**b) c)**

[^177^Lu]Lu-1C1m-Fc conjugated to 3 DOTA gender average human subject dosimetry (mGy/MBq or equivalently Gy/GBq); a) Average Absorbed Dose; b) Lower Absorbed Dose; c) Upper Absorbed Dose values.

Table S6: Target organ AD for the [^64^Cu]Cu-1C1m-Fc 3 DOTA

**a)**


**b) c)**

[^64^Cu]Cu-1C1m-Fc conjugated to 3 DOTA gender average human subject dosimetry (mGy/MBq or equivalently Gy/GBq); a) Average Absorbed Dose; b) Lower Absorbed Dose; c) Upper Absorbed Dose values.
